# Inhibitory Effect of *Moringa oleifera* Seed Extract and Its Behenic Acid Component on *Staphylococcus aureus* Biofilm Formation

**DOI:** 10.3390/antibiotics14010019

**Published:** 2024-12-31

**Authors:** Seoyoung Kim, Tae-Jong Kim

**Affiliations:** Department of Forest Products and Biotechnology, Kookmin University, Seoul 02707, Republic of Korea; tjduddl1231@kookmin.ac.kr

**Keywords:** behenic acid, biofilm, *Moringa oleifera* seed, *Staphylococcus aureus*

## Abstract

**Background/Objectives:** Inhibiting biofilm formation without killing cells facilitates the physical removal of contaminating bacteria while minimizing the opportunity for resistant bacteria to emerge. **Results:** The *M*. *oleifera* methanolic seed extract contained 1.48% behenic acid, significantly inhibiting *S*. *aureus* biofilm formation. Although behenic acid did not affect cell growth, it inhibited biofilm formation in a concentration-dependent manner, up to 20 mg/L. The cell physiology changes caused by behenic acid are potentially unrelated to biofilm formation inhibition, as no correlation was noted between cell hydrophobicity, polysaccharide production, extracellular DNA production, or protein production and behenic acid concentration. Thus, it was hypothesized that the surfactant properties of behenic acid contribute to its ability to inhibit biofilm formation, as a similar biofilm-inhibitory effect was observed when *S. aureus* was administered 1% Tween 80, a surfactant. **Methods**: A methanolic extract of *Moringa oleifera* seeds was selected from a library of edible plant extracts to inhibit *Staphylococcus aureus* biofilm formation without cell killing. **Conclusions**: Behenic acid is a saturated fatty acid that is used as an ingredient in cosmetics and ointments; thus, behenic acid may benefit the skin by inhibiting the biofilm formation of *S*. *aureus*, a commensal skin pathogen.

## 1. Introduction

Biofilms are structured communities of microbial cells that adhere to surfaces and are embedded in a self-produced extracellular polymeric matrix [1]. Biofilms enable bacteria to survive by protecting against environmental stresses, including immune responses and antimicrobial agents [2]. Biofilm formation contributes to chronic infections, medical device contaminations, and the emergence of antibiotic-resistant bacteria, which pose significant challenges to public health and patient recovery [3].

*Staphylococcus aureus* can cause a wide range of infections, from superficial skin conditions to serious conditions such as endocarditis and osteomyelitis [4]. It is a pathogen of particular concern among biofilm-forming pathogens, as the emergence of numerous resistant strains has been reported due to frequent antibiotic exposure [5]. The *S. aureus* biofilm is a critical virulence factor, which enhances its resistance to antibiotics and immune defenses and complicates infection control measures [6]. The extracellular matrix of *S. aureus* biofilms, which consists of polysaccharides, proteins, and extracellular DNA (eDNA), forms a protective barrier that impedes antibiotic penetration [7].

*S. aureus* biofilm formation involves three main stages: initial surface attachment, accumulation and maturation, and dissemination to new niches [8]. Adhesins mediate the initial attachment, while polysaccharide intercellular adhesin (PIA) promotes the accumulation phase [9]. Meanwhile, regulatory systems such as the accessory gene regulator (Agr) and staphylococcal accessory regulator A (SarA) are central to the control of biofilm formation [10]. These mechanisms highlight the complexity of *S. aureus* biofilm development and its contribution to antibiotic resistance.

The increasing prevalence of *S. aureus*-associated infections underscores the urgent need for novel strategies to prevent biofilm formation and eradicate established biofilms. Conventional antibiotics target planktonic cells but are often ineffective against biofilms [11]. Therefore, interest in alternative approaches, such as natural products with biofilm-inhibitory properties, has increased [12,13]. In additional, natural products exhibit antimicrobial activity and provide additional health benefits [14], making them attractive candidates for biofilm inhibition. Unlike bactericidal agents, biofilm inhibitors reduce selective pressure, minimizing the risk of resistance development. This approach facilitates bacterial removal by physical methods, such as washing, without promoting resistance [15].

*Moringa oleifera*, commonly known as the drumstick or miracle tree, is a fast-growing, drought-resistant species of the Moringaceae family. Native to the sub-Himalayan regions of northern India, *M. oleifera* is now widely cultivated in tropical and subtropical areas [16]. Various parts of the *M. oleifera* tree, including the seeds, leaves, and bark, have been traditionally used for their antimicrobial, anti-inflammatory, and antioxidant activities [17,18]. In addition, *M*. *oleifera* seed oil contains oleic, palmitic, and behenic acids and is valued for its nutritional and medicinal benefits, including anticancer, hepatoprotective, and neuroprotective activities [19,20].

Recent studies have highlighted the potential of *M. oleifera* seed extracts as biofilm inhibitors, with components such as flavonoids [21] and lectins [22] showing significant inhibitory activity. Therefore, this study investigated the biofilm-inhibitory effects of *M. oleifera* seed extract and behenic acid, a major extract component. The results show that behenic acid inhibits *S. aureus* biofilm formation without bactericidal effects, offering an alternative strategy to control *S. aureus* infections while minimizing the risk of resistance development.

## 2. Results

### 2.1. Changes in S. aureus Biofilm Formation Induced by M. oleifera Seed Extract

**Figure 1:**
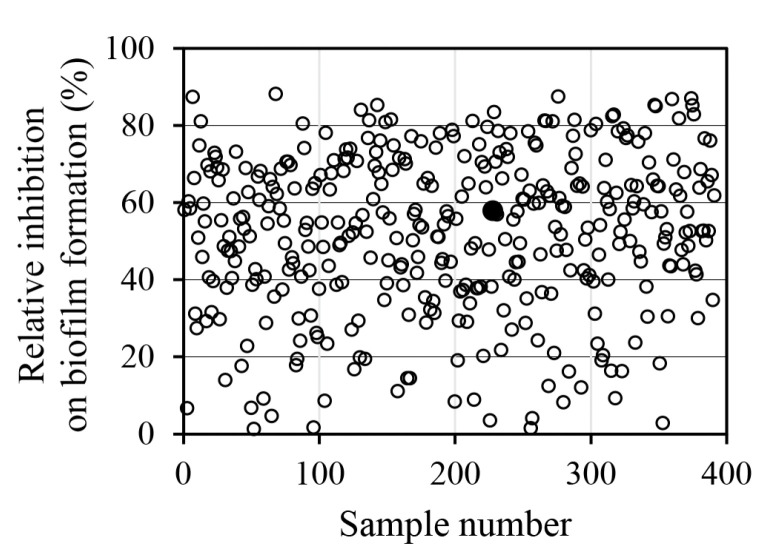
Inhibitory activity of 391 methanolic extracts of edible plants on *S. aureus* biofilm formation. The scientific names of the plants and their relative inhibitory activities are listed in Supplementary Table S1. The closed circle for sample #228 is the methanolic extract of *M*. *oleifera* seed.

**Figure 2:**
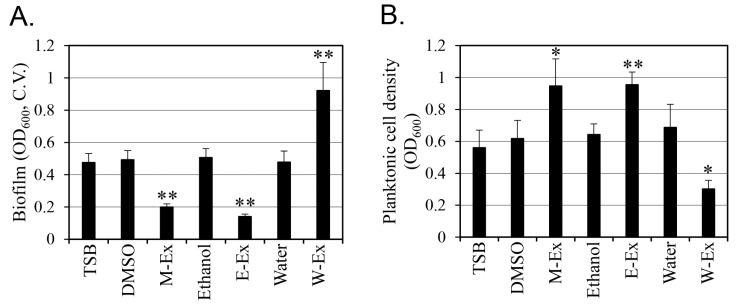
Effects of the *M*. *oleifera* seed extracts on *S. aureus* biofilm formation (**A**) and cell growth (**B**). Biofilm formation was quantified by crystal violet (C.V.) assay. Cell density was observed using optical density (OD) values at 600 nm. TSB: tryptic soy broth; DMSO: TSB culture with 5% dimethyl sulfoxide (DMSO); M-Ex: TSB culture with 20 g/L *M. oleifera* seed methanolic extract dissolved in DMSO; Ethanol: TSB culture with 5% ethanol; E-Ex: TSB culture with 20 g/L *M. oleifera* ethanol seed extract dissolved in ethanol; Water: TSB culture with 5% water; W-Ex: TSB culture with 20 g/L *M. oleifera* seed water extract dissolved in water. Results are presented as the mean value of three replicates with standard deviation. The solvent results of DMSO, ethanol, and water were similar to the TSB results at the 95% confidence level in the *t*-test. The *M*. *oleifera* seed extract results were compared to the solvent alone using a *t*-test. One or two asterisks were placed on the bars when the results differed at the 95% or 99% confidence level, respectively.

A methanolic extract library of 391 edible plants was used to screen for substances that effectively inhibited biofilm formation without killing *S*. *aureus* (Figure 1 and Supplementary Table S1). The *M. oleifera* seed methanolic extract increased *S. aureus* growth by 53% while reducing biofilm formation by 59% (Figure 2). The *M. oleifera* seed ethanol extract showed very similar activity to the methanolic extract, increasing cell growth by 49% and inhibiting biofilm formation by 72%. However, the water extract had the opposite effect, inhibiting cell growth by 56% and increasing biofilm formation by 93%. These contrasting results in cell growth and biofilm formation may be due to the change in the compound composition of the extracts depending on the polarity of the extract solvent.

**Figure 3:**
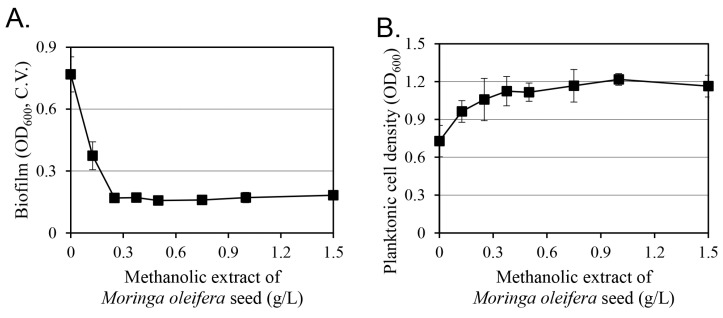
Effect of the *M*. *oleifera* seed methanolic extracts on *S*. *aureus* biofilm formation (**A**) and cell growth (**B**). Biofilm formation was quantified by crystal violet (C.V.) assay. Cell density was observed using optical density (OD) values at 600 nm. Results are presented as the mean value of three replicates with standard deviation. In both figures, the regression analysis showed that the significance *F* was less than 0.01.

To evaluate the efficacy of the *M. oleifera* seed methanolic extract in inhibiting biofilm formation, the concentration-dependent effect of the extract on cell growth and biofilm formation was evaluated (Figure 3). As the methanolic extract concentrations increased (up to 0.25 g/L), biofilm formation decreased in a concentration-dependent manner while cell growth increased. At concentrations above 0.25 g/L, no further changes in cell growth or biofilm formation were observed with increasing concentrations of the *M. oleifera* seed methanolic extract.

*M. oleifera* seeds are rich in lipids and fatty acids, with oleic acid having the highest fatty acid content at 74%, followed by palmitic and behenic acids at approximately about 6% each [23]. The effect of oleic acid [24] and palmitic acid [25] on *S*. *aureus* biofilm formation has already been reported; therefore, this study investigated the effect of behenic acid on *S*. *aureus* biofilm formation. Gas chromatographic analysis of the *M. oleifera* seed methanolic extract produced in this study showed a behenic acid content of 1.48% with a retention time of 21.6–22.1 min (Supplementary Figure S1).

### 2.2. Changes in S. aureus Biofilm Formation by Behenic Acid

**Figure 4:**
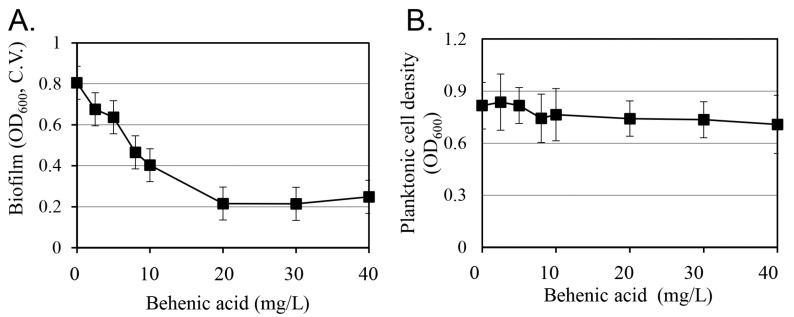
Effect of behenic acid on *S*. *aureus* biofilm formation and cell growth. Biofilm formation (**A**) was quantified by crystal violet (C.V.) assay. Cell density (**B**) was observed using optical density (OD) values at 600 nm. Results are presented as the mean value of three replicates with standard deviation. In both figures, the regression analysis showed that the significance *F* was less than 0.01.

The effect of behenic acid on *S*. *aureus* biofilm formation was evaluated (Figure 4). At 20 mg/L, behenic acid inhibited biofilm formation without affecting cell growth. Biofilm formation decreased in a concentration-dependent manner as the concentration of behenic acid increased, reaching a plateau at 20 mg/L. Furthermore, no change in cell growth was observed up to 40 mg/L, the maximum concentration tested.

**Figure 5:**
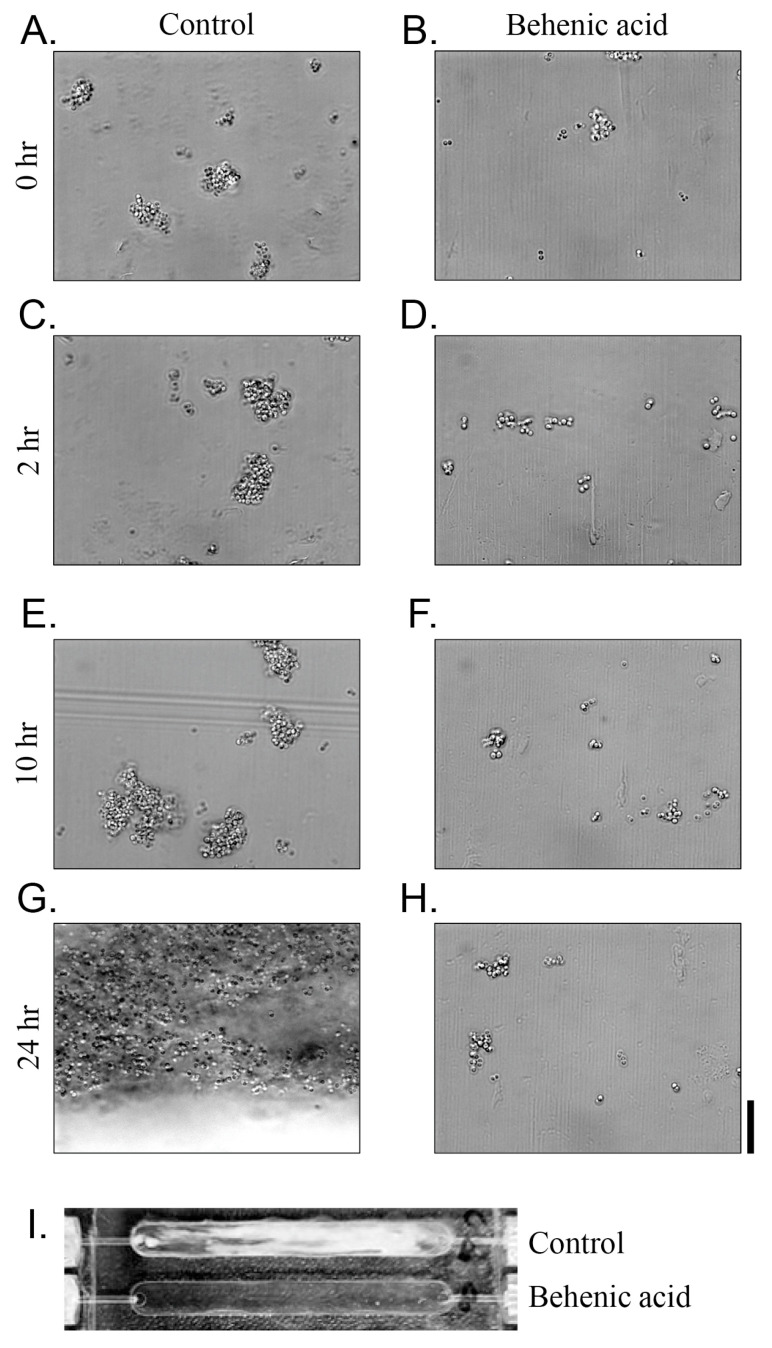
Biofilm formation of *S*. *aureus* in a continuous flow cell culture system. Cells were grown in tryptic soy broth as a control (**A**,**C**,**E**,**G**). Behenic acid (20 mg/L) was added to observe the effect on biofilm formation (**B**,**D**,**F**,**H**). Biofilm formation was observed under a phase-contrast microscope at 1000× magnification from the time of inoculation (0 h; (**A**,**B**)) to 2 h (**C**,**D**), 10 h (**E**,**F**), and 24 h (**G**,**H**). The scale bar at the bottom right indicates 10 μm. Biofilm formation in a non-magnified flow cell is shown in panel (**I**). The white color in the control flow cell indicates the *S*. *aureus* biofilm.

Biofilm formation was evaluated in a continuous flow cell culture system to determine the inhibitory effects of seed extracts (Figure 5). In the control sample without behenic acid, biofilms initially formed in cell clumps and eventually covered all surfaces after 24 h of incubation. However, no cell clumping was observed after incubation with 20 mg/L behenic acid for 24 h, confirming that behenic acid inhibits *S*. *aureus* biofilm formation even in continuous culture. In the microplate experiment, behenic acid did not inhibit cell growth; however, no increase in cell number was observed in the flow cell experiment. Since the cells could not form a biofilm or adhere to the surface in the flow cell, the cells were washed out and no increase in cell number was observed.

### 2.3. Physiological Changes in S. aureus Induced by Behenic Acid

**Figure 6:**
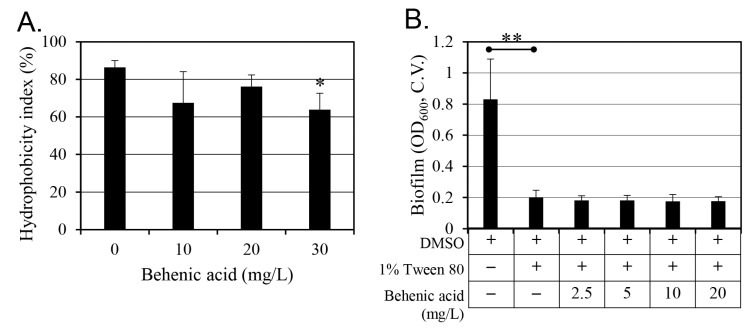
Effect of behenic acid on cell hydrophobicity. In panel (**A**), the changes in cell hydrophobicity induced by behenic acid were evaluated. Results are presented as the mean value of three replicates with standard deviation. Values that differ from the control (0 mg/L behenic acid) with 95% confidence are marked with an asterisk at the top of the bars. In panel (**B**), changes in *S*. *aureus* biofilm formation induced by 1% Tween 80, a surfactant that neutralizes the hydrophobic properties of behenic acid, were evaluated. The amount of biofilm was quantified by crystal violet (C.V.) assay. Results are presented as the mean value of six replicates with standard deviation. The behenic acid result was compared to the second result from the left with 1% Tween 80. Values that differ at the 99% confidence levels are marked with two asterisks.

Cell hydrophobicity strongly influences biofilm formation [26]. Therefore, changes in cell hydrophobicity were measured after behenic acid treatment (Figure 6A). As cell hydrophobicity decreased, biofilm formation on the hydrophobic surface also decreased, while biofilm formation on the hydrophilic surface increased. At 30 mg/L behenic acid, there was a 26% decrease in hydrophobicity; however, no change in hydrophobicity was observed with up to 20 mg/L behenic acid, the concentration that promoted the maximum inhibition of biofilm formation. This observation suggests inconsistent cell hydrophobicity with an inhibitory effect of behenic acid on biofilm formation.

The hydrophobicity of the cells adhering to the hydrophobic surface to which the biofilm is attached can be neutralized by surfactants. This study evaluated the effect of the surfactant, 1% Tween 80, on biofilm formation (Figure 6B). Treatment with 1% Tween 80 alone significantly reduced *S. aureus* biofilm formation. However, the addition of behenic acid did not result in an additional quantitative reduction because 1% Tween 80 had already sufficiently reduced biofilm formation.

**Figure 7:**
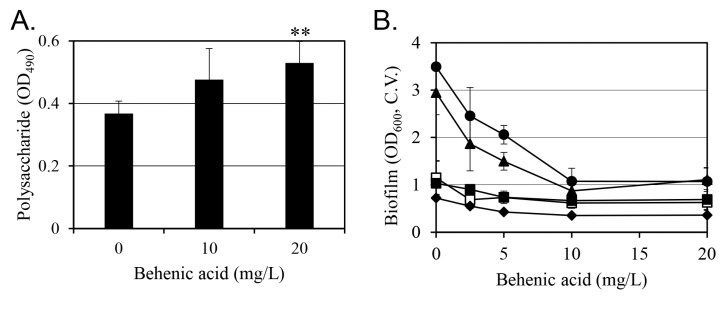
Effect of behenic acid on the amount of polysaccharides. The changes in the polysaccharide amount after behenic acid treatment shown in panel (**A**) are the mean values of four replicates with standard deviation. Values that differ from the control (0 mg/L behenic acid) with 99% confidence are marked with two asterisks at the top of the bars. (**B**) Effect of behenic acid and glucose on biofilm formation of *S. aureus* after 24 h. Biofilm formation was quantified by crystal violet (C.V.) assay. The glucose concentrations were 0% (☐), 0.1% (■), 0.2% (♦), 0.4% (▲), and 0.6% (●). Results are presented as the mean value of five replicates with standard deviation. Regression analysis at 0.1%, 0.4%, and 0.6% glucose concentrations resulted in a significance *F* below 0.05, whereas at 0% and 0.2% glucose, the significance *F* exceeded 0.05.

Microorganisms produce extracellular polymeric substances, such as extracellular DNA (eDNA), proteins, and polysaccharides, for biofilm formation. Therefore, quantitative changes in extracellular polysaccharides induced by behenic acid were evaluated (Figure 7A). Extracellular polysaccharides increased with increasing behenic acid concentrations and were increased by 44% with 20 mg/L behenic acid. In general, an increase in extracellular polysaccharides promotes biofilm formation. Therefore, the increase in extracellular polysaccharides observed after behenic acid treatment does not appear to contribute to the inhibition of biofilm formation. Notably, behenic acid reduced *S. aureus* biofilm formation in a concentration-dependent manner even with glucose, which is known to enhance biofilm formation (Figure 7B). Furthermore, administration of 30 mg/L behenic acid abolished the glucose-induced increase in biofilm formation.

**Figure 8:**
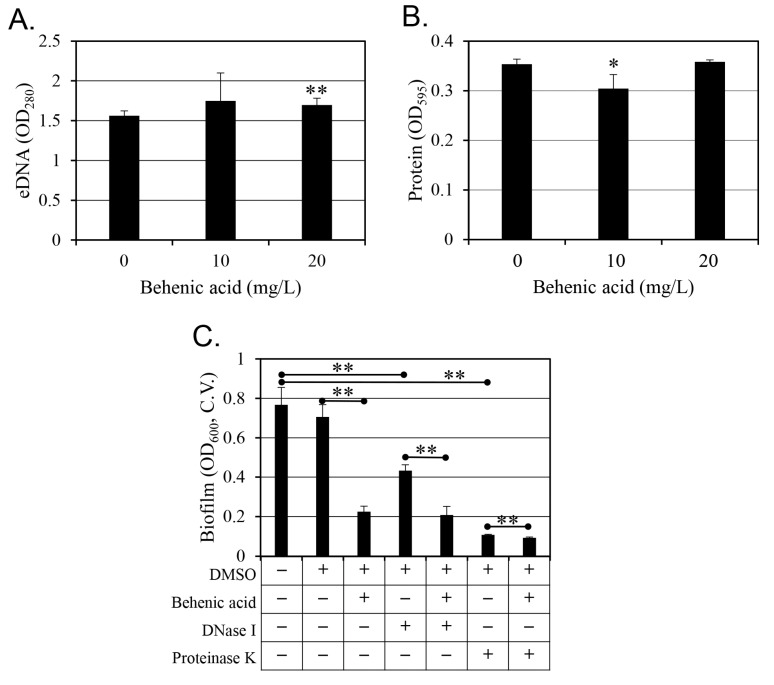
Effect of behenic acid on extracellular DNA (eDNA) and protein concentrations. eDNA concentrations are shown in panel (**A**) as the mean value of eight replicates with standard deviation. Values that differ from the control (0 mg/L behenic acid) at the 99% confidence level are marked with two asterisks at the top of the bars. Changes in protein amount (**B**) are shown as the mean value of four replicates with standard deviation. Values that differ from the control (0 mg/L behenic acid) at the 95% confidence level are marked with an asterisk at the top of the bars. In panel (**C**), to evaluate whether eDNA or protein significantly affect *S*. *aureus* biofilm formation, DNase I, a DNA-degrading enzyme, or proteinase K, a protein-degrading enzyme, was added, and the changes in biofilm formation were evaluated. The concentrations tested were 5% for dimethyl sulfoxide (DMSO), 20 mg/L for behenic acid, 50 U/mL for DNase I, and 0.5 g/L for proteinase K. Biofilm formation was quantified by crystal violet (C.V.) assay. Results are presented as the mean value of six replicates with standard deviation. Values that differ between two results at the 99% confidence level are marked with two asterisks.

After behenic acid treatment, eDNA and extracellular protein changes were also measured (Figure 8A,B respectively). A slight increase in eDNA was observed in response to 20 mg/L behenic acid treatment (Figure 8A). Regarding extracellular proteins, a slight decrease was observed in response to 10 mg/L behenic acid, but no change was observed at 20 mg/L (Figure 8B). These results did not support that the behenic acid-induced inhibition of biofilm formation was related to eDNA and *S*. *aureus* protein changes.

It was hypothesized that if eDNA plays a role in biofilm formation, then since DNase I is a DNA-degrading enzyme, changes in biofilm formation would occur following DNase I treatment [27,28]. Indeed, DNase 1 treatment partially reduced biofilm formation, and behenic acid further enhanced this reduction (Figure 8C). Similarly, it was hypothesized that if extracellular proteins contribute to biofilm formation, since proteinase K is a proteolytic enzyme, then changes in biofilm formation would be observed following proteinase K treatment [29,30]. Indeed, proteinase K further reduced biofilm formation even after the behenic acid-induced reduction. However, because biofilm formation was already significantly reduced by proteinase K, the additional reduction by subsequent behenic acid treatment was limited to 14% (Figure 8C).

In conclusion, behenic acid effectively inhibits *S. aureus* biofilm formation by mechanisms independent of changes in cell hydrophobicity, extracellular polysaccharide production, eDNA levels, or extracellular protein levels. While behenic acid increased extracellular polysaccharides, these changes did not correlate with the inhibitory effect of behenic acid on biofilm formation. In addition, behenic acid neutralized glucose-induced biofilm enhancement, demonstrating that behenic acid has robust anti-biofilm activity. The results from the addition of DNase I and proteinase K suggest that extracellular DNA and proteins partially contribute to biofilm formation but are not the primary targets of behenic acid. These results highlight the unique surfactant-like properties of behenic acid as a key factor in biofilm inhibition and provide a starting point for further exploration of the potential of using behenic acid as a non-bactericidal biofilm inhibitor for clinical and environmental applications.

## 3. Discussion

This study showed that methanol and ethanol extracts of *M. oleifera* seeds significantly inhibited biofilm formation without inhibiting *S*. *aureus* growth, whereas water extracts inhibited cell growth but increased biofilm formation (Figure 2). These results reflect that ethanol and methanol, unlike water, can be used to extract hydrophobic substances, such as behenic acid. In addition, the methanolic extract of *M. oleifera* seeds contained 1.48% behenic acid. In a previous study, the behenic acid content in the *M. oleifera* seed oil extracted with *n*-hexane was about 6% [23]. Hexane, a solvent that is efficient in extracting hydrophobic compounds such as behenic acid, can increase the extraction yield.

Considering the concentration of behenic acid in the extracts, its contribution to biofilm inhibition can be estimated by comparing the most effective biofilm inhibition concentration of behenic acid with that of the extract. Indeed, no further changes in cell growth or biofilm formation were observed at methanolic extract concentrations above 0.25 g/L (Figure 3). In comparison, behenic acid inhibited biofilm formation in a concentration-dependent manner (up to 20 mg/L) but did not affect cell growth (Figure 4). Behenic acid accounted for 1.48% of the *M. oleifera* seed methanolic extract, corresponding to 3.7 mg/L in a 0.25 g/L sample. This amount represents 18.5% of the 20 mg/L behenic acid, where its biofilm-inhibitory effect appeared to be saturated (Figure 4). These results suggest that additional compounds besides behenic acid in the methanolic extract contribute to biofilm inhibition. Nonetheless, the contribution of the 18.5% behenic acid content is likely to be significant in the overall efficacy of *M. oleifera* seed methanolic extract in inhibiting biofilm formation without affecting cell growth.

Surfactants can remove the *S*. *aureus* biofilm [31]. However, since the addition of behenic acid did not further inhibit biofilm formation after 1% Tween 80 treatment alone (Figure 6B), this suggests that Tween 80 saturated the inhibitory effect and could not be further reduced.

Behenic acid is a surfactant (PubChem identifier: CID 8215), which means that it can remove *S*. *aureus* biofilms again. The minimum concentration of 20 mg/L behenic acid that maximally inhibited biofilm formation is much lower than the concentration of other surfactants required to remove *S*. *aureus* biofilms [31,32]. This suggests that behenic acid is an effective compound in inhibiting *S*. *aureus* biofilm formation. In addition, the results indicated that behenic acid-induced changes in the production of extracellular polymeric substances (eDNA, proteins, and polysaccharides) were independent of biofilm formation, supporting the hypothesis that behenic acid inhibits biofilm formation through a surfactant function.

Extracellular polysaccharides are considered an important substance in biofilm formation [33]; however, slime formation by *S*. *aureus* has been reported to be independent of the degree of biofilm formation [34]. Therefore, although polysaccharide production was increased by behenic acid (Figure 7), it is difficult to correlate this observation with the decrease in biofilm formation. In addition, the increased biofilm formation following the addition of glucose to the medium is consistent with previous studies [34,35]. However, this increase was also reduced by behenic acid, with 30 mg/L behenic acid offsetting most of the glucose-induced increase in biofilm formation (Figure 7B), suggesting that behenic acid can eliminate glucose-induced biofilm formation.

In previous studies, the reduced biofilm formation by DNase I treatment (Figure 8C) confirmed the contribution of eDNA to *S*. *aureus* biofilm formation [27,28]. However, 20 mg/L behenic acid caused the same reduction in biofilm formation regardless of DNase I treatment. This suggests that behenic acid treatment may reduce the eDNA contribution to biofilm formation, although the exact mechanism remains unclear. In addition, the biofilm formation was further reduced by the addition of proteinase K compared to the 20 mg/L behenic acid treatment (Figure 8C). Since 20 mg/L behenic acid further reduced biofilm formation beyond the proteinase K-mediated reduction, this suggests that, unlike eDNA, the contribution of proteins to biofilm formation is independent of behenic acid, and its effect is additive.

Future research should focus on elucidating the precise mechanisms by which behenic acid and other components of *M. oleifera* seed extracts inhibit biofilm formation and explore their efficacy against other pathogenic biofilms. In addition, further investigations to optimize extraction methods and evaluate the therapeutic potential of these compounds in clinical settings are warranted.

## 4. Materials and Methods

### 4.1. Strain and Chemicals

*Staphylococcus aureus* ATCC 6538 was purchased from the Korean Collection for Type Cultures (Jeongeup, Republic of Korea). The cells were stored at −80 °C with 25% glycerol. Tryptic soybean broth (TSB, catalog number: 211825) and Bacto agar (catalog number: 214010) were purchased from Becton Dickinson Korea Co., Ltd., (Seoul, Republic of Korea). Tryptic soybean agar (TSA) was prepared using TSB with 1.5% Bacto agar. Phosphate-buffered saline (PBS) was prepared using 8 g/L sodium chloride, 0.2 g/L potassium chloride, 1.44 g/L sodium phosphate dibasic, and 0.245 g/L potassium phosphate monobasic. Dimethyl sulfoxide (DMSO, catalog number: 000D0458) and Tween 80 (catalog number: 000T0900) were purchased from Samchun Chemicals Co., Ltd., (Seoul, Republic of Korea). Behenic acid (catalog number: B1747) was purchased from Tokyo Chemical Industry Co., Ltd., (Tokyo, Japan). DNase I (catalog number: 79254) was purchased from QIAGEN Korea Ltd., (Seoul, Republic of Korea). Proteinase K (Lot No. 14C11PO3) was purchased from Biofact Co., Ltd., (Daejeon, Republic of Korea).

### 4.2. Extract Preparation of M. Oleifera Seeds

*M. oleifera* seeds were purchased from the Jiundang Oriental Pharmacy (Seoul, Republic of Korea), and the extract was prepared according to a previous study [36]. Briefly, extraction was performed on 30 g of *M. oleifera* seeds with diameters of 3 mm or less using 300 mL of methanol (catalog number: 000E1095; Samchun Chemical Co., Ltd.), 95% ethyl alcohol (catalog number: 000E0219; Samchun Chemical Co., Ltd.), or water at 50 °C for 3 h. Next, the extraction solution was filtered using Whatman^TM^ qualitative filter paper, grade 1 (catalog number: 1002-110; Cytiva^TM^, Sigma-Aldrich, St. Louis, MO, USA), to remove particles. Afterward, the filtrate was concentrated using a rotary evaporator (RV-10; IKA Korea, Seoul, Republic of Korea), and the concentrate of water extract only was lyophilized using a lyophilizer (FDU-2110; EYELA, Seongnam, Republic of Korea) for 3 days. Finally, the extracts were stored at −80 °C.

### 4.3. Culture Condition for S. aureus

Following storage at −80 °C, *S*. *aureus* was streaked onto TSA plates and incubated at 37 °C for 24 h. Single colonies were inoculated in 5 mL of TSB and incubated at 37 °C for 24 h. Then, pre-cultured cells were inoculated in 20 mL of TSB in a 250 mL baffled flask at a concentration with an OD_600_ of 0.1 and incubated at 37 °C for 24 h. To measure cell growth, either the cell density was measured using an absorbance of 600 nm, or the live cell density was measured by counting colony-forming units (CFUs). To calculate the CFUs, the cell cultures were serially diluted 10-fold, and then 100 μL of these diluted cultures was spread on TSA plates. After incubation at 37 °C for 24 h, the number of colonies on the plates was counted. The number of colonies per volume was calculated from the number of colonies on the plate, which ranged from 25 to 250 colonies per plate, considering the dilution factor.

### 4.4. Quantitative Analysis of Biofilm Formation by S. aureus

Biofilm formation was evaluated according to previous studies [13,37]. Briefly, quantitative analysis of biofilm formation was performed using 96-well polyvinyl chloride (PVC) microplates (catalog number: 2797; Corning Korea Company Ltd., Seoul, Republic of Korea). Pre-cultured cells were inoculated into 96-well PVC plates containing 100 µL TSB in each well to a final concentration of 2.0 × 10^7^ CFU/mL. Then, the inoculated microplates were incubated at 37 °C for 24 h.

The biofilm amount was measured using 1% crystal violet according to the method used in a previous study [17]. Briefly, the solution was removed from the cultured 96-well PVC microplates and washed three times with distilled water. The crystal violet (1%, 100 µL) was added to each well and incubated at 23 °C for 15 min, and the wells were again rinsed three times with distilled water. The crystal violet retained in the biofilm was eluted using 100 µL of 95% ethanol at 23 °C for 15 min. Afterward, the biofilms were quantitatively assessed by measuring the absorbance of the eluted crystal violet at 600 nm using a Synergy™ LX Multimode Reader (BioTek Instruments Korea Ltd., Seoul, Republic of Korea). The degree of biofilm formation inhibition was determined by comparing the absorbance of the biofilm formed with the sample to the maximum absorbance of the biofilm formed without the sample. The relative decrease in absorbance was determined as the inhibitory effect of the sample.

### 4.5. Quantitative Analysis of Behenic Acid in M. oleifera Seed Extracts Using Gas Chromatography

Behenic acid in the *M. oleifera* seed extract was quantitatively analyzed using a modified version of the MIDI method [38]. Briefly, cells were harvested, lyophilized, saponified, and methylated following a previously reported protocol. Next, fatty acids were extracted using 1.25 mL of hexane/methyl-*tert*-butyl ether (1:1 *v*/*v*), washed with 3 mL of 0.3 M NaOH, and analyzed using gas chromatography (model: 7890B; Agilent Technologies, Inc., Wilmington, DE, USA) with a flame ionization detector and an HP-5 column (CN: 19091J-413; Agilent Technologies). The initial oven temperature of 70 °C was maintained for 1 min, then increased by 6.25 °C/min to 325 °C, and maintained for 3.6 min. The carrier gas, nitrogen, was delivered at a 0.9 mL/min flow rate. The sample (1 µL) was injected at a 10:1 split ratio. The injector and detector temperatures were set to 230 °C and 280 °C, respectively. Behenic acid was identified using external standards, and its concentration in the *M. oleifera* methanolic seed extracts was determined using a standard curve.

### 4.6. Formation of Biofilms in Continuous Flow Cell Culture

*S*. *aureus* biofilm formation in a flow cell, a continuous-culture device, was induced according to previous studies [39,40]. Flow cells (model number: ACCFL0001) were purchased from IBI Scientific (Dubuque, IA, USA). Bubble traps (catalog number: Z741751) were purchased from Merck KGaA (Darmstadt, Germany). The chamber was sealed with Rinzl plastic coverslips (catalog number: 72261-50; Electron Microscopy Sciences, Hatfield, PA, USA). TSB was used for continuously feeding the medium by a peristaltic pump (model: MFLX07522-20; VWR International, LLC., Radnor, PA, USA) equipped with a cartridge pump 8 (model: 7519-25; VWR International, LLC.). The entire system was sterilized by 0.5% (*v*/*v*) sodium hypochlorite at 3 mL/h for 4 h. Afterward, distilled water was flushed through at 3 mL/h for 18 h to remove all sodium hypochlorite in the system.

The cells were cultured in TSB at 37 °C for 24 h and then diluted to an OD_600_ of 0.1 using TSB. Next, a 350 μL aliquot of the diluted culture was inoculated into the flow cell, and the cells were allowed to attach for 1 h, after which the flow was initiated at 0.5 mL/min [41]. Biofilm pictures were taken at the indicated time points with phase-contrast microscopy using an Axio Scope microscope equipped with an Axiocam camera (Carl Zeiss Microscopy GmbH, Gottingen, Germany). The biofilm was finally observed at 400×.

### 4.7. Measurement of Cell Hydrophobicity

Cell hydrophobicity was measured with modifications from previous studies [37,42]. The pre-cultured cells were inoculated to an OD_600_ of 0.1 in 1 mL of TSB. Next, the substance to be tested was added according to the conditions and incubated at 37 °C at 250 rpm for 24 h. Then, the cells were harvested by centrifugation at 7280× *g* for 10 min. After washing twice with PBS, the cells were resuspended in 4 mL of PBS, and the OD_600_ (*A***_0_**) was measured. Subsequently, 0.4 mL of n-hexadecane was added, and the mixture was vortexed for 1 min and incubated at 23 °C for 15 min. The aqueous layer was taken from the two separated layers, and the OD_600_ (*A*) was measured. The hydrophobicity index (HI) was subsequently calculated from the measured value. The formula is as follows:

HI (%) = [(*A***_0_**−*A*)/*A***_0_**] ✕ 100

### 4.8. Quantitative Analysis of Exopolysaccharide (EPS)

A quantitative analysis of EPS was performed according to previous studies [37,43]. First, behenic acid was mixed with 5 mL of TSB and inoculated into 2.0 × 10^7^ CFU/mL of the pre-cultured cells. After incubation at 37 °C and 250 rpm for 6 h, the first cell-free supernatant was collected by centrifugation at 4300× *g* for 10 min and then stored at −80 °C. Next, the cell pellet was rinsed with 5 mL of PBS, mixed with 5 mL of isotonic buffer (10 mM Tris/HCl at pH 8.0, 10 mM EDTA, and 2.5% NaCl), and incubated at 4 °C for 12 h. After vigorous mixing for 3 min, the second cell-free supernatant was collected by centrifugation at 4300× *g* for 15 min. Then, the first and second cell-free supernatants were mixed, and ice-cold ethanol was added to three times the volume of the mixture. Next, the mixture was incubated at −20 °C for 12 h. Finally, the dried EPS was obtained after centrifugation at 4300× *g* for 15 min and dried at 23 °C.

The EPS proteins were analyzed using the Bradford method [44]. Briefly, 100 µL of EPS dissolved in water was mixed with 1 mL of Bradford reagent (Biosesang, Seongnam, Republic of Korea) and incubated at 23 °C for 2 min. Then, the absorbance was measured at 595 nm.

According to a previous study, the polysaccharides in the EPS were analyzed using phenol–sulfuric acid [45]. Briefly, 200 µL of the EPS dissolved in water and 600 µL of sulfuric acid were vigorously mixed. Next, phenol (5%, 120 µL) was added, and the mixture was incubated at 23 °C for 10 min before the absorbance was measured at 490 nm.

A spectrophotometer analyzed the eDNA of the EPS. First, the EPS was dissolved in 1 mL of water, and the absorbance was measured at 260 nm and 280 nm. Afterward, the eDNA concentration was calculated using the Abs_260_/Abs_280_ ratio.

### 4.9. Statistical Analysis

The values of the solvent-treated control and the substance-treated experimental groups were compared using the *t*-test (Excel, Microsoft) to analyze statistical significance. The corresponding results show the group comparison test and the level set for significant differences.

## 5. Conclusions

*M*. *oleifera* seed methanolic extracts effectively inhibit biofilm formation by *S*. *aureus* without affecting bacterial growth, with behenic acid identified as the active component responsible for this effect. The surfactant properties of behenic acid likely contribute to its biofilm-inhibitory activity, consistent with the established role of fatty acids as surfactants capable of disrupting biofilm structures. Behenic acid is commonly used in cosmetics as a thickener, lubricant, and opacifier, and the results of this study highlight its additional potential to inhibit *S*. *aureus* biofilm formation. While these results suggest the utility of behenic acid as a targeted approach to prevent bacterial biofilm formation on the skin, further studies are needed to substantiate its broader applications and mechanisms in clinical settings.

## 6. Patents

Composition for preventing biofilm formation comprising behenic acid, Korean Patent Registration number: 10-2659787. 2024.04.18.

## Data Availability

The original contributions presented in the study are included in the article/Supplementary Materials. Further inquiries can be directed to the corresponding author.

## Supplementary Materials

The following supporting information can be downloaded at https://www.mdpi.com/article/10.3390/antibiotics14010019/s1, Table S1: Inhibitory activity of plant methanolic extracts on *Staphylococcus aureus* biofilm formation; Figure S1: Gas chromatography analysis of behenic acid in the methanolic *M*. *oleifera* seed extract.
