# Supplementary material for: Inhibitory Effect of Moringa oleifera Seed Extract and Its Behenic Acid Component on Staphylococcus aureus Biofilm Formation
Source: Antibiotics (Basel). 2024 Dec 31;14(1):19. doi: 10.3390/antibiotics14010019 (PMC11762368; doi:10.3390/antibiotics14010019)
Supplement: Supplementary file 1 [file antibiotics-14-00019-s001.zip › antibiotics-3364587-supplementary.pdf]

Figure S1. Gas chromatography analysis of behenic acid in the methanolic *M. oleifera* seed extract.

A. Gas chromatography analysis of the methanolic *M. oleifera* seed extract alone. The X-axis unit is minutes, and the retention time of behenic acid is 21.691 minutes.

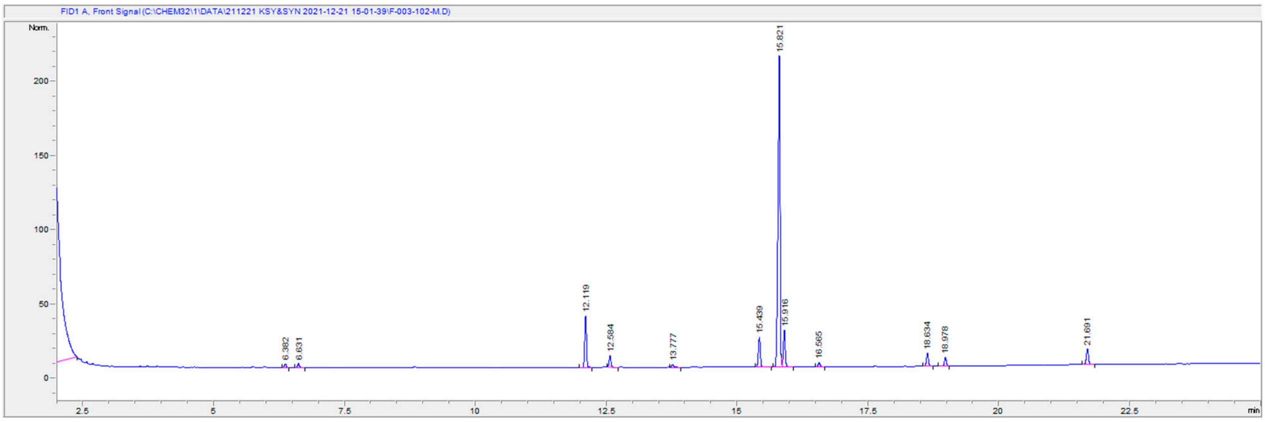

#	Time	Type	Area	Height	Width	Area%	Symmetry
1	1.632	BB S	854784.4	398438.5	0.034	99.896	0.344
2	6.382	BB	5.6	2.5	0.0349	0.001	1.02
3	6.631	BB	6.4	2.5	0.0385	0.001	0.861
4	12.119	BB	80.3	34.4	0.0362	0.009	1.078
5	12.584	BB	18.8	7.9	0.0377	0.002	0.995
6	13.777	BB	5	1.9	0.0409	0.001	0.876
7	15.439	BB	52.5	20.2	0.0413	0.006	1.059
8	15.821	BV R	583.8	209.2	0.0425	0.068	1.736
9	15.916	VB E	61.1	24.4	0.0382	0.007	1.054
10	16.565	BB	7.7	3	0.0398	0.001	0.917
11	18.634	BB	22.6	8.9	0.0396	0.003	1.015
12	18.978	BB	15.6	5.8	0.0414	0.002	1.115
13	21.691	BB	30.7	10.7	0.0455	0.004	1.029

B. Gas chromatography analysis of the methanolic *M. oleifera* seed extract with 3.5 g/L of behenic acid. The blue line represents the gas chromatography analysis of the methanolic *M. oleifera* seed extract alone, while the red line shows the results of the extract with behenic acid. The X-axis unit is minutes.

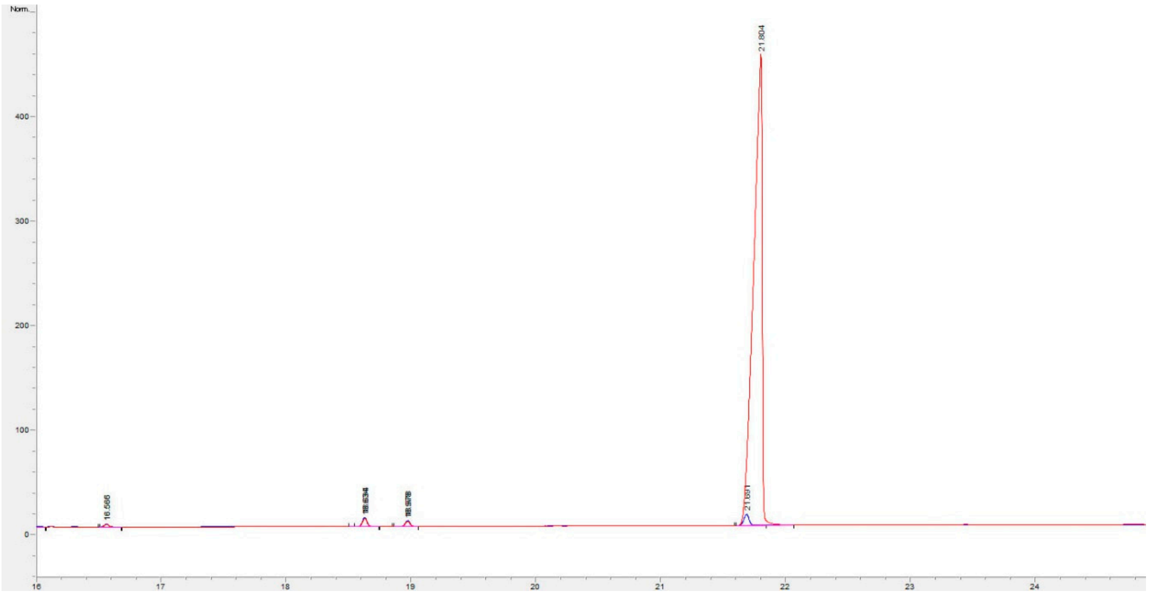

C. Gas chromatography analysis for the standard curve of behenic acid. The blue, red, and green lines represent behenic acid concentrations of 0.96 g/L, 1.99 g/L, and 3.00 g/L, respectively. The X-axis unit is minutes.

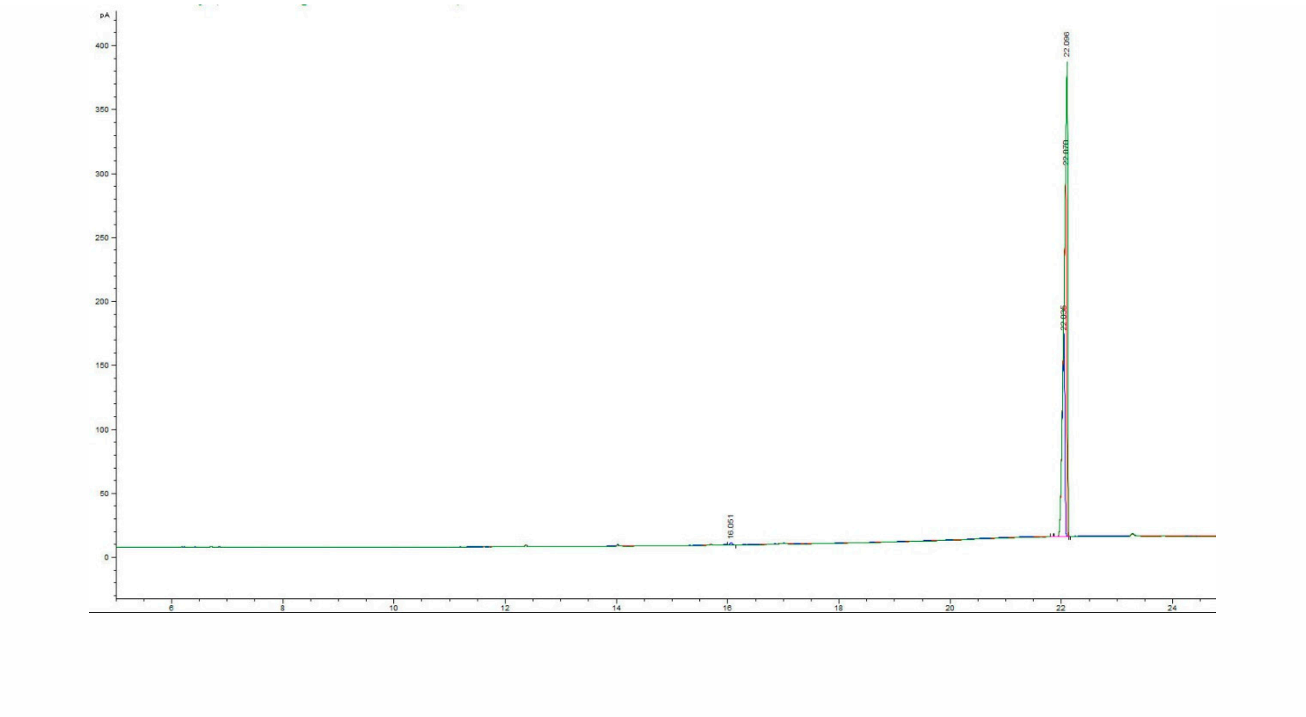

D. Standard curve for the quantitative analysis of behenic acid. The X-axis unit is mg/L of behenic acid.

The equation for the standard curve is as follows:

$$A = 0.5729695 \times C_B - 11.808009$$

where A is the area value from the gas chromatography analysis, and C_B is the concentration of behenic acid (mg/L).

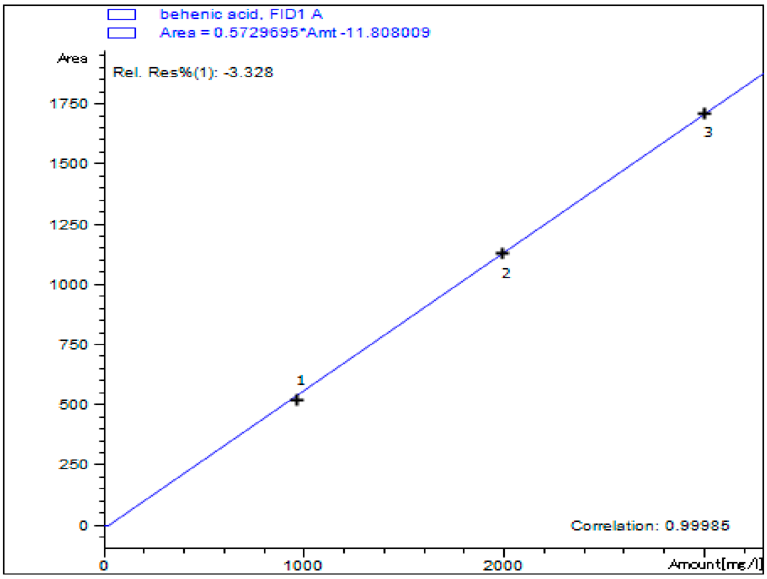

Table S1. Inhibitory activity of plant methanolic extracts on *Staphylococcus aureus* biofilm formation.

No.	Scientific name of plant	Name of herbal medicine	Name of the plant part	Relative inhibition on biofilm formation (%)
1	*Acanthopanax sessiliflorum*	Acanthopanax Root Bark		58.1
2	*Achyranthes japonica*	Achyranthis Radix		-0.6
3	*Aconitum carmichaeli*	Aconiti Lateralis Radix Preparata		6.7
4	*Aconitum carmichaeli*	Aconiti Tuber		60.2
5	*Aconitum kusnezoffii*	Aconiti Kusnezoffii Tuber		58.5
6	*Acorus gramineus*	Acori Gramineri Rhizoma		-25.7
7	*Actinidia arguta*	Actinidiae Fructus		87.4
8	*Actinidia chinensis*		Kiwi	66.3
9	*Actinidia polygama*	Actnidiae Polygammae Fructus		31.2
10	*Adansonia spp.*		Fruit	27.4
11	*Adenophora triphylla*	Adenophorae Radix		50.8
12	*Agastache rugosa*	Agastachis Herba		74.8
13	*Agrimonia pilosa*	Agrimoniae Herba		81.1
14	*Ailanthus altissima*	Ailanthi Radicis Cortex		45.9
15	*Akebia quinata*	Akebiae Caulis		59.7
16	*Alisma orientale*	Alismatis Rhizoma		55.1
17	*Allium cepa*			29.3

18	*Allium fistulosum*	Allii Fistulosi Bulbus		69.7
19	*Allium fistulosum*		Large green onion	40.6
20	*Allium sativum*		Garlic	68.1
21	*Allium tuberosum*	Alli Tuberosi Semen		31.6
22	*Allium tuberosum*		Chives	39.6
23	*Aloe bera*		Aloe	72.8
24	*Alpinia katsumadai*	Alpiniae Katsumadai Semen		71.8
25	*Alpinia officinarum*	Alpiniae Officinari Rhizoma		69.1
26	*Alpiniae Fructus*	Alpinia oxyphylla Miq.		65.8
27	*Amaranthus caudatus*		Flower	29.8
28	*Amomum kravanh*	Amomi Fructus Rotundus		55.4
29	*Amomum tsao-ko*	Amomi Tsao-ko Fructus		68.5
30	*Anacardium occidentale*		Cashew nut	48.6
31	*Anemarrhena asphodeloides*	Anemarrhenae Rhizoma		14.0
32	*Anethum graveolens*	Anethi Fructus		37.9
33	*Anethum graveolens*		Seed	47.6
34	*Angelica dahurica*	Angelicae Dahuricae Radix		51.1
35	*Angelica gigas*	Angelicae Gigantis Radix		47.5
36	*Angelica keiskei*	Angelicae Herba		40.4
37	*Annona muricata*		Graviola	61.1
38	*Apium graveolens*	Apii Herba		44.9
39	*Arachis hypogaea*	Arachidis Semen		73.2
40	*Aralia continentalis*	Araliae Continentalis Radix		-11.7

41	*Arctium lappa*	Arctii Semen		48.6
42	*Areca catechu*	Arecae Pericarpium		55.8
43	*Areca catechu*	Arecae Semen		17.6
44	*Arisaema amurense*	Arisaematis Rhizoma		56.2
45	*Aronia arbutifolia*		Aronia berry	53.2
46	*Artemisia annua*	Artemisiae Annuae Herba		68.9
47	*Artemisia argyi*	Artemisiae Argyi Herba		22.8
48	*Artemisia capillaris*	Artemisiae Capillaris Herba		62.7
49	*Asiasarum heterotropoides*	Asiasari Radix et Rhizoma		51.3
50	*Asparagus cochinchinensis*	Asparagi Tuber		6.8
51	*Aster tataricus*	Asteris Radix et Rhizoma		38.7
52	*Astragalus membranaceus*	Astragali Radix		1.3
53	*Atractylodes japonica*	Atractylodis Rhizoma Alba		42.7
54	*Atractylodes lancea*	Atractylodis Rhizoma		40.1
55	*Aucklandia lappa*	Aucklandiae Radix		66.7
56	*Avena sativa*			60.7
57	*Benincasa cerifera*	Benincasae Pericarpium	Seed	68.2
58	*Benincasa cerifera*	Benincasae Pericarpium	Skin of seed	-218.1
59	*Beta vulgaris*		Root fruit	9.2
60	*Beta vulgaris*		Chard	40.8
61	*Boesenbergia pandurata*		Root	28.8

62	*Brassica oleracea*		Aerial part	54.6
63	*Brassica oleracea*		Cabbage	59.1
64	*Brassica oleracea*		Kale	66.0
65	*Brassica rapa*		Pak choi	4.7
66	*Buddleja officinalis*	Buddlejae Flos		64.0
67	*Bupleurum falcatum*	Bupleuri Radix		35.6
68	*Caesalpinia sappan*	Sappan Lignum		88.1
69	*Camellia sinensis*		Leaf	62.2
70	*Canavalia gladiata*	Canavaliae Semen		-11.4
71	*Capsicum*		Paprika (orange)	58.5
72	*Capsicum*		Paprika (yellow)	68.8
73	*Carthamus tinctorius*	Carthami Flos		37.4
74	*Carthamus tinctorius*	Carthami Fructus		55.3
75	*Carum carvi*			49.4
76	*Carya illinoinensis*		Pecan nut	70.6
77	*Cassia angustifolia*	Sennae Folium		70.6
78	*Cassia tora*	Cassiae Semen		42.6
79	*Castanea crenata*	Castaneae Semen		69.7
80	*Celosia argentea*	Celosiae Semen		45.8
81	*Chaenomeles sinensis*	Chaenomelis Fructus		44.2
82	*Chelidonium majus*	Chelidonii Herba		63.6
83	*Chlorella spp.*		Cell	17.8
84	*Chrysanthemum morifolium*	Chrysanthemi Flos		19.5

85	*Cibotium barometz*	Cibotii Rhizoma		30.0
86	*Cicer arietinum*		Seed	24.1
87	*Cimicifuga heracleifolia*	Cimicifugae Rhizoma		40.8
88	*Cinnamomum cassia*	Cinnamomi Ramulus		80.5
89	*Cinnamomum cassia*	Cinnamomi Cortex Spissus		74.2
90	*Cinnamomum cassia*	Cinnamomi Cortex		52.9
91	*Cirsium japonicum*	Cirsii Radix		54.7
92	*Cirsium setidens*		Thistle	48.6
93	*Cistanche deserticola*	Cistanchis Herba		42.5
94	*Citrus aurantium*	Aurantii Fructus Immaturus		30.7
95	*Citrus junos*		Citron	63.6
96	*Citrus reticulata*	Aurantii Pericarpium		1.7
97	*Citrus unshiu*	Citri Unshiu Semen		65.0
98	*Citrus unshiu*	Citri Unshius Pericarpium Immaturus		26.2
99	*Clematis manshurica*	Clematidis Radix		25.2
100	*Clerodendron trichotomum*	Clerodenri Trechotomi Folium		37.7
101	*Cnidium monieri*	Cnidi Fructus		67.1
102	*Cnidium officinale*	Cnidii Rhizoma		54.8
103	*Cocculus trilobus*	Cocculi Radix		48.5
104	*Cocos nucifera*		Coconut	8.6
105	*Codonopsis lanceolata*	Codonopsidis Radix		78.1
106	*Codonopsis pilosula*	Codonopsis Pilosulae Radix		23.4

107	*Coffea arabica*		Coffee	43.6
108	*Coix lacryma-jobi*	Coicis Semen		63.3
109	*Commiphora myrrha*	Myrrha		67.6
110	*Coptis japonica*	Coptidis Rhizoma		-170.8
111	*Corchorus olitorius*		Leaf	70.9
112	*Cordyceps militaris*		Militaris anthelmintic plant	-34.8
113	*Coriandrum sativum*		Seed	38.7
114	*Coriolus versicolor*	Coriolus Polyporus		54.8
115	*Cornus officinalis*	Corni Fructus		48.9
116	*Corydalis ternata*	Corydalis Tuber		49.5
117	*Crataegus pinnatifida*	Crataegi Fructus		39.3
118	*Cucucrbita maxima*		Autumn squash	68.1
119	*Cucumis melo*	Melonis Pedicellus		71.4
120	*Cucurbita moschata*		Pumpkin seed	73.8
121	*Cucurbita moschata*		Pumpkin	71.6
122	*Cuminum cyminum*		Seed	51.4
123	*Curcuma longa*	Curcumae Longae Rhizoma		74.0
124	*Curcuma phaeocaulis*	Zedoariae Rhizoma		27.0
125	*Cuscuta chinensis*	Cuscutae Semen		52.3
126	*Cynanchum wilfordii*	Cynanchi Wilfordii Radix		16.8
127	*Cynomorium songaricum*	Cynomorii Herba		54.6
128	*Cyperus rotundus*	Cyperi Rhizoma		70.8

129	*Daucus carota*		Purple carrot	29.4
130	*Daucus carota subsp. Sativus*		Carrot	19.9
131	*Dendrobium nobile*	Dendrobii Herba		84.0
132	*Dictamnus dasycarpus*	Dictamni Radicis Cortex		56.8
133	*Dimocarpus longan*	Longanae Arillus		-3.6
134	*Dioscorea batatas*	Dioscoreae Rhizoma		19.5
135	*Dioscorea batatas*		Root fruit	52.4
136	*Diospyros kaki*		Leaf	76.7
137	*Diospyros kaki*	Kaki Calyx	Tip of fruit	81.4
138	*Dolichos lablab*	Dolichoris Semen		45.7
139	*Dopspyros lotus*		Seed	-3.3
140	*Drynaria fortunei*	Drynariae Rhizoma		60.9
141	*Dryopteris crassirhizoma*	Crassirhizomae Rhizoma		69.5
142	*Duchesnea indica*	Duchesneae Herba		73.1
143	*Eclipta prostrata*	Ecliptae Herba		85.3
144	*Elsholtzia ciliata*	Elsholtziae Herba		67.8
145	*Ephedra sinica*	Ephedrae Herba		76.1
146	*Epimedium koreanum*	Epimedii Herba		64.8
147	*Equisetum arvense*	Equiseti Herba		57.5
148	*Equisetum hyemale*	Equiseti Herba		34.7
149	*Eriobotrya japonica*	Eriobotryae Folium		80.9
150	*Eucommia ulmoides*		Leaf	39.1
151	*Eucommia ulmoides*	Eucommiae Cortex	Bark	45.1

152	*Euonymus alatus*	Euonymi Lignum Suberalatum		55.9
153	*Euryale ferox*	Euryales Semen		81.6
154	*Evodia rutaecarpa*	Evodiae Fructus		68.4
155	*Fagopyrum esculentum*		Seed	74.8
156	*Fagopyrum tataricum*		Fagopyrum tataricum	-292.2
157	*Ficus carica*	Fici Fructus		50.8
158	*Foeniculum vulgare*	Foeniculi Fructus		11.1
159	*Forsythia viridissima*	Forsythiae Fructus		71.5
160	*Fragaria × ananassa*		Strawberry	43.2
161	*Fritillaria thunbergii*	Fritillaria Thunbergii Bulb		44.0
162	*Gadus chalcogrammus*		Dried pollack	38.5
163	*Ganoderma lucidum*	Ganoderma		71.3
164	*Gardenia jasminoides*	Gardeniae Fructus		70.1
165	*Gastrodia elata*	Gastrodiae Rhizoma		14.5
166	*Gentiana macrophylla*	Gentianae Macrophyllae Radix		30.9
167	*Gentiana scabra*	Gentianae scabrae Radix		14.5
168	*Geranium thunbergii*	Geranii Herba		77.2
169	*Ginkgo biloba*	Ginkgonis Semen		50.2
170	*Ginkgo biloba Linné*	Ginkgo Folium		57.0
171	*Glebionis coronaria*		Crown daisy	58.1
172	*Glechoma hederacea*	Glechomae Herba		41.7
173	*Glycine max*	Glycine Semen Germinatum		45.9
174	*Glycine max*		Bean sprouts	54.1
175	*Glycyrrhiza uralensis*	Glycyrrhizae Radix et Rhizoma		75.8

176	*Gossypium herbaceum*	Gossypii Semen		53.6
177	*Helianthus annuus*	Helianthis Semen		64.9
178	*Helianthus tuberosus*			35.4
179	*Hericium erinaceus*			28.9
180	*Hibiscus*		Flower leaf	66.3
181	*Hijikia fusiforme*	Sargassum		-20.8
182	*Hordeum vulgare*	Hordei Fructus Germinatus		32.3
183	*Houttuynia cordata*	Houttuyniae Herba		64.3
184	*Hovenia dulcis*	Hoveniae Semen seu Fructus		34.5
185	*Ilex paraguayensis*		Leaf	31.4
186	*Illicium verum*	Illici Veri Fructus		74.2
187	*Impatiens balsamina*	Impatientis Semen		51.1
188	*Imperata cylindrica*	Imperatae Rhizoma		51.1
189	*Inula japonica*	Inulae Flos		77.9
190	*Ipomoea batatas*	Ipomoeae Tuba		44.4
191	*Ipomoea batatas*		Sweet potato	45.3
192	*Isatis indigotica*	Isatidis Folium		54.3
193	*Isatis indigotica*	Isatidis Radix		39.7
194	*Ixeris dentata*	Ixertis Herba		57.7
195	*Kalopanax pictus*	Kalopanacis Cortex		56.4
196	*Kochia scoparia*	Kochiae Fructus		-178.0
197	*Lactuca sativa L*		Blue lettuce	44.7
198	*Laurus nobilis*	Lauri Fructus		78.8
199	*Lavandula species*		Flower	77.2

200	*Lens culinaris*		Lentil	8.4
201	*Lentinula edodes*		Shiitake	55.8
202	*Leonurus japonicus*	Leonuri Herba		19.0
203	*Lepidium apetalum*	Lepidii seu Descurainiae Semen		29.4
204	*Ligusticum tenuissimum*	Angelicae Tenuissimae Radix		37.0
205	*Ligustrum lucidum*	Ligustri Fructus		61.6
206	*Lilium lancifolium*	Lilii Bulbus		37.4
207	*Lindera strichnifolia*	Linderae Radix		72.0
208	*Linum usitatissimum*	Lini Semen		38.7
209	*Liriope platyphylla*	Liriopis Tuber		29.0
210	*Lithospermum erythrorhizon*	Lithospermi Radix		64.9
211	*Lonicera japonica*	Lonicerae Flos		33.9
212	*Lonicera japonica*	Lonicerae Folium		48.1
213	*Loranthus parasticus*	Visci Herba et Loranthi Ramulus		81.2
214	*Luffa cylindrica*	Luffae Fructus Retinervus		8.9
215	*Lycium chinense*		Leaf	49.4
216	*Lycium chinense*	Lycii Fructus	Seed	37.8
217	*Lycium chinense*	Lycii Radicis Cortex		37.8
218	*Lycopus lucidus*	Lycopi Herba		75.1
219	*Magnolia denudata*	Magnoliae Flos		38.2
220	*Magnolia ovobata*	Magnoliae Cortex		70.5

221	*Malus domestica*		Fruit	20.2
222	*Malva verticillata*	Malvae Semen		69.3
223	*Mentha arvensis*	Menthae Herba		63.9
224	*Momordica charantia*			79.6
225	*Morinda citrifolia*			47.8
226	*Morinda officinalis*	Morindae Radix		3.6
227	*Moringa oleifera*		Leaf	38.2
**228**	***Moringa oleifera***		**Seed**	**57.8**
229	*Morus alba*	Mori Cortex Radicis	Root bark	83.4
230	*Morus alba*	Mori Fructus	Fruit	70.6
231	*Morus alba*	Mori Folium	Leaf	56.9
232	*Morus alba*	Mori Ramulus	Branch	78.5
233	*Myrciaria dubia*		Fruit	73.0
234	*Myristica fragrans*	Myristicae Semen		21.8
235	*Myristica fragrans*		Nutmeg	66.2
236	*Mytilus coruscus*		Mussel	32.0
237	*Nasturtium officinale*		Watercress	50.5
238	*Nelumbo nucifera*	Nelumbinis Semen		73.9
239	*Nelumbo nucifera*	Nelumbinis Folium		71.9
240	*Nelumbo nucifera*	Nelumbinis Rhizomatis nodus		40.8
241	*Nelumbo nucifera*		Lotus root	78.0
242	*Ocimum basilicum*	Ocimi Herba		27.0
243	*Oenanthe javanica*		Water parsley	55.6
244	*Oenothera biennis*			40.0

245	*Oenothera fruticosa*		Seed	44.5
246	*Opuntia ficus-indica*		Cactus fruit	57.7
247	*Opuntia humifusa*		Leaf	44.6
248	*Origanum majorana*		Leaf	49.4
249	*Origanum vulgare*		Leaf	67.2
250	*Oryza sativa*		Rice bran	61.0
251	*Oscillatoria spirulina*		Cell	60.8
252	*Ostericum koreanum*	Osterici Radix		28.7
253	*Paeonia lactiflora*	Paeoniae Radix Alba		35.1
254	*Paeonia lactiflora*	Paeoniae Radix Rubra		78.5
255	*Paeonia suffruticosa*	Moutan Cortex Radicis		63.1
256	*Panax ginseng*	Ginseng Radix		1.5
257	*Panax noto-ginseng*	Notoginseng Radix		4.1
258	*Panicum miliaceum*		Millet	59.7
259	*Patrinia scabiosifolia*		Seed	75.4
260	*Perilla frutescens*	Perilla Herba		74.8
261	*Perilla frutescens*	Perilliae Semen		24.2
262	*Perilla frutescens*	Perillae Japonicae Semen	Perilla	59.9
263	*Perilla frutescens*		Perilla leaf	46.5
264	*Persea americana*		Avocado	36.8
265	*Petroselinum crispum*		Parsley	64.4
266	*Pharbitis nil*	Pharbitidis Semen		81.2
267	*Phellinus igniarius*	Phellini Polyporus		81.1
268	*Phellodendron*	Phellodendri Cortex		62.9

	amurense			
269	*Phlomis umbrosa*	Phlomidis Radix		12.4
270	*Phragmites communis*	Phragmitis Rhizoma		61.8
271	*Phyllostachys nigra*		Leaf	36.4
272	*Phyllostachys nigra*	Phyllostachyos Caulis in Taeniam		81.1
273	*Pimpinella brachycarpa*		Leaf	21.0
274	*Pinus densiflora*	Pini Pollen	Pollen	53.7
275	*Pinus densiflora*		Leaf	47.5
276	*Pinus densiflora*		Succinum	87.5
277	*Piper nigrum*	Pireris Nigri Fructus		61.1
278	*Pistacia vera*		Pistachio	51.7
279	*Plantago asiatica*	Plantaginis Herba		59.1
280	*Platycodon grandiflorum*	Platycodonis Radix		8.2
281	*Polygala tenuifolia*	Polygalae Radix		58.9
282	*Polygonatum humile*	Polygonati Rhizoma		47.7
283	*Polygonatum robustum*	Polygonati Odorati Rhizoma		-1.3
284	*Polygonatum sibiricum*	Polygonati Rhizoma		16.2
285	*Polygonatum stenophyllum*	Polygonati Rhizoma		42.4
286	*Polygonum aviculare*	Polygoni Avicularis Herba		69.0
287	*Polygonum cuspidatum*	Polygoni Cuspidati Rhizoma et Radix		77.3

288	*Polygonum multiflorum*	Polygoni Multiflori Radix		81.5
289	*Polyporus umbellatus*	Polyporus		72.6
290	*Poncirus trifoliata*	Ponciri Fructus Immaturus		64.4
291	*Ponus sylvestris*		Pine needle	-35.8
292	*Poria cocos*	Poria(Hoelen)		64.9
293	*Portulaca oleracea*	Portulacae Herba		12.1
294	*Prunella vulgaris*	Prunellae Spica		64.1
295	*Prunus armeniaca*	Armeniacae Semen		42.4
296	*Prunus dulcis*			50.4
297	*Prunus japonica*	Pruni Nakaii Semen		40.3
298	*Prunus mume*	Mume Fructus		53.5
299	*Prunus persica*	Persicae Semen		41.2
300	*Psidium guajava*	Psidii Folium et Fructus		78.7
301	*Psoralea corylifolia*	Psoraleae Semen		-374.2
302	*Pteridum aquilinum*		Bracken	39.6
303	*Pueraria lobata*	Puerariae Radix		31.1
304	*Pueraria lobata*	Puerariae Flos		80.4
305	*Pulsatilla koreana*	Pulsatillae Radix		23.4
306	*Quercus acutissima*		Acorn	46.4
307	*Raphanus sativus*	Raphani Semen		54.2
308	*Raphanus sativus*		Radish	19.1
309	*Rehmannia glutinosa*	Rehmanniae Radix Siccus		20.4
310	*Rhaponticum uniflorum*	Echinopsis Radix		63.7
311	*Rheum palmatum*	Rhei Radix et Rhizoma		71.1

312	*Ribes nigrum*		Blackcurrant	60.2
313	*Ricinus communis*	Ricini Semen		40.1
314	*Rosa laevigata*	Rosae Laevigatae Fructus		58.3
315	*Rosa multiflora*	Rosae Fructus		16.4
316	*Rosa multiflora*	Rosae Multiflorae Flos		82.4
317	*Rosmarinus officinalis*		Leaf	82.8
318	*Rubus coreanus*	Rubi Fructus		9.3
319	*Rubus spp.*		Blackberry	62.5
320	*Rumex japonicus*	Rumecis Radix		78.4
321	*Saccharina japonica*		Kelp	49.3
322	*Salicornia herbacea*	Salicorniae Herba		52.4
323	*Salvia miltiorrhiza*	Salviae Miltiorrhizae Radix		16.3
324	*Salvia officinalis*		Leaf	79.2
325	*Sambucus spp.*		Elderberry	55.6
326	*Sambucus williamsii*	Sambuci Lignum		76.7
327	*Sanguisorba officinalis*	Sanguisorbae Radix		77.4
328	*Santalum album*	Santali Albi Lignum		-2.5
329	*Saposhnikovia divaricata*	Saposhnikoviae Radix		50.0
330	*Sarcodon imbricatus*		Carpophore	64.5
331	*Schisandra chinensis*	Schisandrae Fructus		58.5
332	*Schizonepeta tenuifolia*	Schizonepetae Spica		60.3
333	*Scrophularia buergeriana*	Scrophulariae Radix		23.7
334	*Scutellaria baicalensis*	Scutellariae Radix		64.2

335	*Scutellaria barbata*	Scutellariae Barbatae Herba		75.8
336	*Secale cereale*		Rye	47.3
337	*Siegesbeckia pubescens*	Siegesbeckiae Herba		44.7
338	*Silybum marianum*		Milk thistle	-79.0
339	*Sinapis alba*	Sinapis Semen		59.6
340	*Smilax china*	Smilacis Rhizoma		78.0
341	*Solanum*		Fruit	38.2
342	*Solanum nigrum*		Leaf	30.4
343	*Sophora flavescens*	Sophorae Radix		70.4
344	*Sophora japonica*	Sophorae Fructus		-20.2
345	*Sophora japonica*	Sophorae Flos		57.5
346	*Sorbus commixta*	Sorbi Lignum et Fructus		65.9
347	*Sorbus commixta*		Bark	85.3
348	*Sorghum bicolor*		Sorghum	85.0
349	*Spatholobus suberectus*	Spatholobi Caulis		64.4
350	*Spinacia oleracea*		Spinach	64.2
351	*Spirodela polyrrhyza*	Spirodelae Herba		18.4
352	*Stachys riederi*	Stachytis Herba		57.7
353	*Stellaria dichotoma*	Stellariae seu Gypsophilae Radix		2.9
354	*Stevia rebaudiana*		Leaf	49.3
355	*Syzygium aromaticum*	Syzygii Flos		51.0
356	*Tagetes spp.*		Flower	53.1
357	*Taraxacum coreanum*		Dandelion root	30.5

358	*Taraxacum platycarpum*	Taraxaci Herba		43.6
359	*Taraxacum platycarpum*		Dandelion flower	43.4
360	*Terminalia chebula*	Terminaliae Fructus		86.8
361	*Theobroma cacao*		Cocoa	71.1
362	*Thuja orientalis*	Thujae Semen		-27.9
363	*Thymus vulgaris*	Thymi Herba		63.4
364	*Torreya nuncifera*	Torreyae Semen		-14.3
365	*Trapa bispinosa*	Trapae Fructus		81.8
366	*Tremella fuciformis*		Carpophore	61.8
367	*Tribulus terrestris*	Tribuli Fructus		47.7
368	*Tricholoma matsutake*	Tricholomae Polyporus		43.9
369	*Trichosanthes kirilowii*	Trichosanthis Radix		67.9
370	*Trichosanthes kirilowii*	Trichosanthis Semen		52.2
371	*Trigonella foenum-graecum*	Trigonellae Semen		57.7
372	*Tussilago farfara*	Farfarae Flos		48.8
373	*Typha orientalis*	Typhae Pollen		52.6
374	*Ulmus parvifolia*		Root bark	87.0
375	*Ulmus pumila*	Ulmi Cortex		85.2
376	*Uncaria sinensis*	Uncariae Ramulus Et Uncus		82.9
377	*Undaria pinnatifida*		Sea mustard	42.4
378	*Vaccinium myrtillus*		Fruit	41.4
379	*Valeriana fauriei*	Valerianae Radix et Rhizoma		30.0
380	*Vigna angularis*		Adzuki beans	63.8

381	*Vigna radiatus*	Vignae Radiatae Semen		68.6
382	*Viola mandshurica*	Violae Herba		52.6
383	*Viscum album*	Visci Herba		52.7
384	*Vitex rotundifolia*	Viticis Fructus		76.7
385	*Xanthium strumarium*	Xanthii Fructus		50.2
386	*Zanthoxylum piperitum*	Zanthoxyli Fructus		65.5
387	*Zea mays*	Maydis Stigma		52.6
388	*Zingiber officinale*	Zingiberis Rhizoma		76.1
389	*Zizania latitolia*		Breakwater plant	67.1
390	*Zizyphus jujuba*	Zizyphi Fructus		34.8
391	*Zizyphus jujuba*	Zizyphi Semen		61.8
